# Multicenter Randomized Trial to Prevent Pulmonary Vein Stump Thrombus After Left-Sided Anatomical Lung Resection

**DOI:** 10.1016/j.atssr.2025.11.026

**Published:** 2025-12-19

**Authors:** Aritoshi Hattori, Kazuya Takamochi, Keisuke Ariyoshi, Sachiyo Furumi, Shunsuke Oyamada, Kenji Suzuki

**Affiliations:** 1 Department of General Thoracic Surgery, Juntendo University School of Medicine, Tokyo, Japan; 2 Department of Data Management, Japanese Organisation for Research and Treatment of Cancer Data Center, Tokyo, Japan; 3 Department of Biostatistics, Japanese Organisation for Research and Treatment of Cancer Data Center, Tokyo, Japan

## Abstract

**Background:**

Pulmonary vein stump thrombosis (PVST) is frequently detected on follow-up computed tomography in patients who have undergone left-sided anatomical pulmonary resection. In particular, a floating thrombus in the pulmonary vein stump has been identified as a potential cause of several postoperative systemic embolic complications, including cerebral infarction, highlighting the unmet need to improve postoperative outcomes in general thoracic surgery.

**Methods:**

This randomized trial aims to confirm the efficacy of postoperative heparin administration in preventing PVST and postoperative cerebral infarction, focusing exclusively on left-sided anatomical pulmonary resections. The primary end point is the frequency of PVST on contrast-enhanced computed tomography scans at postoperative day 5 ± 2. The secondary end point is the frequency of systemic thromboembolic events, including cerebral infarction. The study expects to enroll 250 patients from 26 institutions over 2.5 years.

**Results:**

The PV STUDY (Postoperative heparin administration to prevent pulmonary vein stump thrombus after left side anatomical pulmonary resection: Multicenter Randomized Phase II trial) has been open since October 2024, and the trial registration is now ongoing.

**Conclusions:**

The primary analysis is scheduled for spring 2027.

## Visual Abstract

**Figure:**
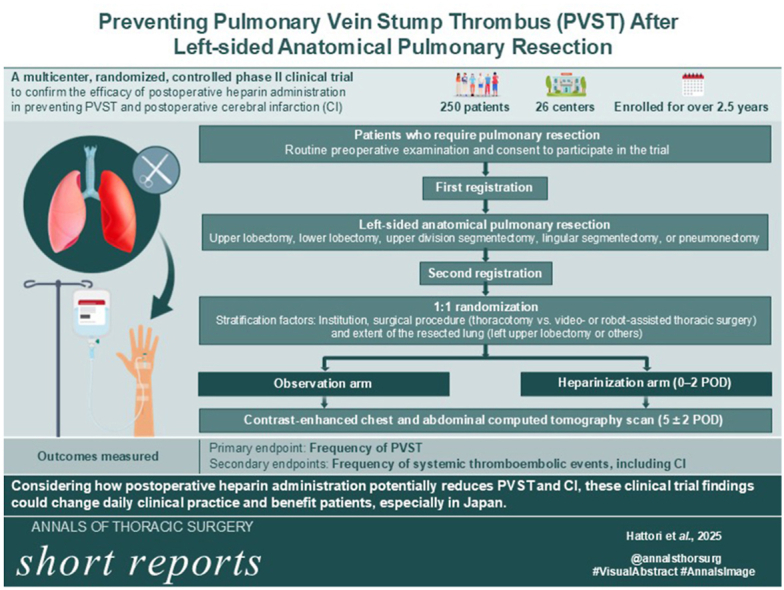

**In Short**

▪ This randomized trial is planned to confirm the efficacy of postoperative heparin administration to prevent pulmonary vein stump thrombus and postoperative cerebral infarction, exclusively focusing on the left-side anatomical pulmonary resection.
▪ The primary end point is the frequency of pulmonary vein stump thrombus by contrast-enhanced computed tomography scan. Secondary end points were the frequency of systemic thromboembolic events, including cerebral infarction.

Postoperative cerebral infarction (CI) is widely recognized as a fatal complication of pulmonary resection. Although the incidence of postoperative CI has been reported to be relatively low, occurring in ∼1% to 2% of general thoracic operations, its clinical course is often devastating and sometimes fatal.^1^, ^2^, ^3^ Therefore, an unmet need exists for strategies to prevent postoperative CI in daily practice. Recently, pulmonary vein stump thrombus (PVST), which is frequently detected on follow-up computed tomography (CT) scans, has become well recognized, and several reports have demonstrated a significant incidence of PVST after left-sided anatomical pulmonary resection, especially left upper lobectomy (LUL).^1^^,^^4^, ^5^, ^6^, ^7^ Importantly, concerns have grown regarding this issue, because a floating thrombus in the pulmonary vein stump after lobectomy may lead to postoperative CI. In particular, PVST after LUL has been identified as a potential cause of several systemic embolic complications.^1^^,^^4^, ^5^, ^6^, ^7^

However, the etiology and risk factors for thrombus formation after pulmonary resection have not yet been clearly identified because all previous studies were retrospective. To address these issues and to explore strategies for preventing postoperative CI after general thoracic surgery, a multicenter prospective observational study was conducted in Japan in 2018 to evaluate the incidence of stump thrombus, clinical characteristics, and risk factors for thrombus formation using postoperative contrast-enhanced CT scans.^8^

Multivariable analysis showed that LUL, or left-sided pulmonary resection, was independently associated with thrombus formation, accounting for >30% of cases, especially after LUL.^8^ Furthermore, PVST developed very early after left-sided pulmonary resection, particularly within the first 1 to 2 weeks.^8^ In addition, this prospective study demonstrated that postoperative CI occurring within 1 week was significantly associated with left-sided anatomical pulmonary resection.^8^

To date, the causes of postoperative CI after lobectomy and its correlation with floating thrombi in the pulmonary vein stump have not been fully clarified. However, given the high frequency of thrombus formation associated with the surgical procedure in daily practice, great caution should be taken when monitoring patients for PVST after left-sided pulmonary resection, because this condition may underlie systemic postoperative thromboembolic disease, including CI.

On the basis of these findings, we designed this randomized phase 2 trial to confirm the efficacy of postoperative heparin administration in preventing PVST and postoperative CI, focusing exclusively on left-sided anatomical pulmonary resection.

## Patients and Methods

### Protocol Digest of The Study

#### Purpose

We will conduct a randomized phase 2 trial, PV STUDY (Postoperative heparin administration to prevent pulmonary vein stump thrombus after left side anatomical pulmonary resection: Multicenter Randomized Phase II trial) to confirm the efficacy of postoperative heparin administration in preventing PVST after left-sided anatomical pulmonary resection.

#### Study Setting

The PV STUDY is a multi-institutional (26 specialized centers) randomized controlled phase 2 trial (Institutional Review Board number: J23-005, the Japan Registry of Clinical Trials number: jRCT1031230739).

#### End Points

The primary end point is the incidence of PVST after left-sided anatomical pulmonary resection. The presence or absence of PVST will be confirmed by contrast-enhanced thoracoabdominal CT scan at postoperative day (POD) 5 ± 2 (ie, POD 3-7). Secondary end points are the frequency of systemic thromboembolic events, including CI in the early phase after surgical resection (within 30 days after surgery); the percentage of PVST shrinkage; frequency of residual PVST; systemic thromboembolic events, including CI, in the late phase after surgical resection (3, 6, and 12 months after surgery); safety analysis for postoperative heparin administration (duration of chest tube drainage, bleeding-related complications, reinsertion of chest drainage tube); respiratory-related complications; and postoperative 30-day and 90-day mortality rates. Adverse events will be evaluated according to the Common Terminology Criteria for Adverse Events, version 5.0 (United States Department of Health and Human Services), and the Japan Clinical Oncology Group postoperative complication criteria, based on the Clavien-Dindo classification.

### Inclusion Criteria

#### First Registration Inclusion Criteria

1. Age 18 to 85 years on the day of registration.
2. LUL, left lower lobectomy, left-sided lobectomy with an additional segmentectomy or wedge resection of the other lobe, pneumonectomy, left upper division segmentectomy, and lingular segmentectomy planned.
3. No history of previous anatomical thoracic surgery on the left side.
4. Performance status of Eastern Cooperative Oncology Group 0 or 1.
5. The latest blood examination result within 30 days before the first registration meeting all of the following:
  a. White blood cell count ≥3.0 × 10^3^/mm^3^ and ≤12.0 × 10^3^/mm^3^
  b. Hemoglobin ≥8.0 g/dL
  c. Platelet count ≥100 × 10^3^/mm^3^
  d. Total bilirubin ≤2.0 mg/dL
  e. Aspartate transferase ≤100 U/L
  f. Alanine aminotransferase ≤100 U/L
  g. Serum creatinine ≤133 μmol/L (1.5 mg/dL)
  h. Normal activated partial thromboplastin time (APTT) (≤1.2-fold of control)
6. No previous history of severe bronchial asthma.
7. No history of allergy to contrast agent.
8. Written consent to participate in the study obtained from the patient.

#### Second Registration Inclusion Criteria

1. LUL, left lower lobectomy, left-sided lobectomy with additional segmentectomy or wedge resection of the other lobe, pneumonectomy, left upper division segmentectomy, and lingular segmentectomy performed.
2. No aggravation of hematoma on the postoperative chest radiography.
3. Serosanguinous fluid from the chest drainage tube.
4. No aggravation of subcutaneous emphysema or continuous air leak requiring reoperation.

### Exclusion Criteria

Patients who meet any of the following criteria will be excluded from this trial:

#### First Registration Exclusion Criteria

1. History of heparin hypersensitivity or history of thrombocytopenia.
2. History of heart failure, unstable angina (angina that developed or worsened within the last 6 months), or myocardial infarction within 6 months.
3. History of CI.
4. Poorly controlled diabetes mellitus (urinary glucose ≥10 g/d or fasting blood glucose ≥8.3 mmol/L (150 mg/dL) under insulin use).
5. Need for preoperative heparinization to replace preoperative antiplatelet or anticoagulant drug administration.
6. Receiving continuous systemic administration (oral or intravenous) of steroids.
7. History of diseases with bleeding tendency or coagulation abnormality.
8. History of treatment for refractory ulcer.
9. Pregnant or lactating women.
10. Patients with psychiatric disorders or psychiatric symptoms that interfere with daily life and make participation in the study difficult.
11. Patients judged unsuitable for enrollment in this study by the research director.

#### Second Registration Exclusion Criteria

1. Surgical resection of pericardium, diaphragm, or chest wall.
2. Bronchoplastic procedure or pulmonary vessel reconstruction.
3. Intrapericardial procedure for pulmonary vein resection.
4. Complete or extensive adhesion in the thoracic cavity.
5. Patients judged unsuitable for enrollment in this study by the research director.

### Randomization

After the first registration criteria are confirmed, registration will be performed using the Viedoc web-based system (Viedoc Technologies). After confirmation of the second inclusion criteria, patients will be randomized (1:1) to the observation or heparinization arms using the stratified permuted block randomization method to balance the following stratification factors: institution, surgical procedure (thoracotomy vs video- or robot-assisted thoracic surgery), and extent of the resected lung (LUL or others).

### Treatment Methods

In the observation arm, standard postoperative management will be administered after pulmonary resection. In the heparinization arm, postoperative heparinization will be started within 2 hours after thoracic surgery and will be performed by intravenous injection using a precision drip. An initial dose of heparin will be calculated based on the patient’s body weight (200 U/kg/d). Heparinization will be continued until POD 2, and APTT will be monitored daily to determine the proper heparin dosage, aiming for 1.5-fold of the control value. The increase or decrease in heparin dosage will be based on the conversion table of heparin administration in the guidelines for the management of pulmonary thromboembolism, deep venous thrombosis, and pulmonary hypertension. In both arms, contrast-enhanced thoracoabdominal CT scan will be performed within POD 5 ± 2 to confirm the presence or absence of PVST. If PVST is confirmed, the use of anticoagulant drugs will be recommended, but the decision will remain at the discretion of each participating institution, because there is no clear evidence supporting anticoagulant treatment of PVST. The overall trial scheme is illustrated in the Figure.

**Figure.**
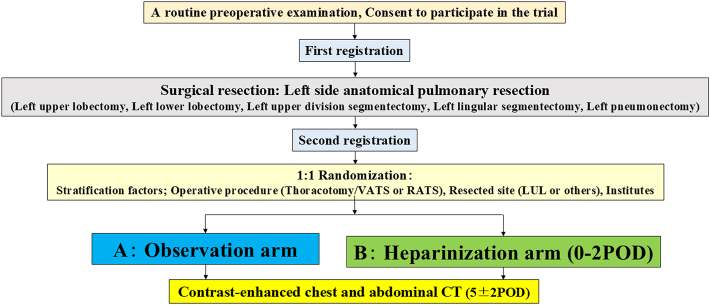
Schema of clinical trials of the postoperative heparin administration to prevent pulmonary vein stump thrombus after left side anatomical pulmonary resection: Multicenter Randomized controlled trial (PV STUDY). (CT, computed tomography; LUL, left upper lobe; POD, postoperative day; RATS, robot-assisted thoracoscopic surgery; VATS, video-assisted thoracoscopic surgery.)

### Follow-Up

All randomized patients will be monitored for at least 1 year after the completion of patient accrual. Contrast-enhanced thoracoabdominal CT will be performed at 3, 6, and 12 months after termination of the treatment protocol.

### Study Design and Statistical Analysis

For our sample size estimation, we used a 2-sample test to determine the difference in proportions between treatment arms. We estimated that a sample size of 250 patients would provide 80% power to detect a 14% reduction in the presence of PVST in the heparinization arm compared with that in the observation arm (ranging from 27% in patients receiving standard observational therapy, considered the null hypothesis, to 13% in patients receiving postoperative heparinization^8^), with a 2-sided type I error of 5%. All statistical analyses will be performed at the Japanese Organisation for Research and Treatment of Cancer (JORTC) Data Center.

### Data Collection, Data Management, and Central Monitoring

The electronic data capture system will be used for randomization, data entry, data management, and central monitoring. The JORTC Data Center will conduct data management and central monitoring to ensure data quality, evaluate study progress, and ensure participant safety. Interim analyses or audits are not planned.

## Results

The PV STUDY has been open since October 2024, and the trial registration is now ongoing.

## Comment

Information from this prospective observational study on the characteristics of PVST^8^ could contribute to a paradigm shift in postoperative management after left-sided pulmonary resection. In this previous prospective observational study, however, a remaining issue is that early-phase CT scanning to detect PVST could not reduce the incidence of postoperative CI because the sudden onset of CI occurred much earlier after pulmonary resection. Furthermore, if a floating thrombus in the pulmonary vein stump is the cause of CI, a postoperative contrast-enhanced CT scan after the onset of CI may not detect PVST because the thrombus may detach and migrate to systemic organs, including the brain. Once postoperative thromboembolic morbidities, including CI, develop, both the clinical course and prognosis are poor.^9^^,^^10^ Hence, in this prospective randomized trial, we plan to use postoperative heparinization within 2 hours after left-sided pulmonary resection to prevent PVST and subsequent CI as effectively as possible, strictly monitoring the APTT value. We believe that quick start of anticoagulation therapy would contribute to the reduction of PVST.

With regard to the duration of heparinization, we planned to use heparin for 3 days (0-2 POD), to not only interrupt the general postoperative course but also enhance the clinical effect in this trial. Furthermore, if the PVST is found by the postoperative contrast-enhanced CT scan, additional anticoagulation is recommended based on the surgeon’s discretion in this study. In general, however, pharmacologic prophylaxis for thromboembolisms is not routinely performed after the lung resection in many Asian countries, which may be due to the difference of coagulation ability among the nations. Actually, it would also be a nationwide trend in Japan. Hence, if the result of this study is positive, the clinical impact to use heparin for preventing PVST is immense in daily practice.

Meanwhile, a clear treatment algorithm for the detected PVST is lacking all over the world, because there is no consensus on the optimal management for the detected PVST. In addition, the rate of postoperative CI is ∼0.5% for right-side lung resection, whereas that of the left-side is 1.5%, which is accounted for 3-times higher in the left-side lung resection.^8^ If the PVST contributes to the postoperative CI at the very early phase after lung resection, we considered that postoperative heparinization should be started soon after the very early phase after lung resection to reduce the rate of PVST by half as a clinically meaning value in this trial.

In conclusion, there are many unmet needs for CI prevention in the postoperative management of general thoracic surgery. This multicenter randomized phase 2 trial in Japan aims to assess the clinical benefits of pharmacologic thromboembolic prophylaxis for PVST and CI after left-sided pulmonary resection.
